# The mediating role of caffeine and biological age in the association between dietary index for gut microbiota and osteoporosis

**DOI:** 10.3389/fnut.2025.1559674

**Published:** 2025-06-13

**Authors:** Yaxiong Li, Hong Cao, Jingyuan Zhang

**Affiliations:** ^1^Department of Spine, Renmin Hospital, Hubei University of Medicine, Shiyan, China; ^2^Department of Traumatic Orthopedics, Renmin Hospital, Hubei University of Medicine, Shiyan, China

**Keywords:** NHANES, dietary index for gut microbiota (DI-GM), osteoporosis, caffeine, biological age

## Abstract

**Background:**

The Dietary Index for Gut Microbiota (DI-GM) is a novel metric developed to evaluate the diversity of intestinal microbiota. However, its relationship with osteoporosis remains uncertain.

**Methods:**

This study utilized data from the National Health and Nutrition Examination Survey (NHANES) conducted between 2007 and 2018. The DI-GM score was derived from two 24-h dietary recall interviews, while bone mineral density (BMD) was measured using dual-energy X-ray absorptiometry (QDR 4500A). Osteopenia and osteoporosis were diagnosed according to the World Health Organization (WHO) criteria. Age-standardized incidence rates (ASIRs) were calculated through direct standardization to the 2,000 U. S. standard population. Additionally, the study employed multivariate logistic regression, restricted cubic spline (RCS) analysis, mediation analysis, and subgroup analysis to explore the data comprehensively.

**Results:**

Weighted logistic regression analysis revealed that higher DI-GM scores were significantly negatively associated with the risk of osteoporosis. Compared to the Q1 group, the Q4 group exhibited a significantly reduced risk of osteoporosis (OR = 0.781, 95% CI: 0.693–0.869). RCS curve analysis identified a nonlinear relationship between DI-GM and osteoporosis, with a critical inflection point at 3.9. Mediation analysis demonstrated that Phenotypic Age (PA), Klemera-Doubal Method (KDM) and caffeine mediated 4.73, 4.55, and 20.33% of the association between DI-GM and osteoporosis, respectively. Furthermore, age-standardized incidence rate analysis showed that the ASIR of osteoporosis was highest among women aged 60–79 years (65.09%). The ASIR for Non-Hispanic Black individuals was significantly lower compared to other racial groups.

**Conclusion:**

Higher DI-GM scores were associated with a reduced risk of developing osteoporosis, with biological age and caffeine serving as mediators in this relationship.

## Introduction

With the acceleration of global population aging, osteoporosis has emerged as a significant public health challenge worldwide (1). According to data from the World Health Organization (WHO), the number of people affected by osteoporosis reached 300 million in 2020, and this figure is projected to rise further as the population continues to age (2). Osteoporosis and osteopenia are among the leading causes of fractures from mechanical forces in individuals over 50 years of age, second only to falls (3). In Canada, over 57,413 patients are hospitalized annually for osteoporosis-related fractures, resulting in more than $1.2 billion in emergency care costs and a total economic burden exceeding 2.3 billion Canadian dollars per year (4). In Western countries, one in three women and one in five men over the age of 50 will experience an osteoporotic fracture during their lifetime (5, 6). Moreover, reduced bone density has been positively associated with increased arterial wall thickness (7). Low bone density is also closely linked to cognitive impairments, including Alzheimer’s disease and mild cognitive impairment (8, 9). Therefore, early intervention in osteoporosis management is essential for reducing both the disease and economic burdens.

Coffee is one of the most widely consumed beverages worldwide. In general, coffee consumption may offer significant benefits for inflammatory diseases and the nervous system due to its caffeine content and other bioactive compounds, such as phenolic acids and diterpenoids (e.g., cafestol and kahweol) (10). Several systematic reviews have suggested that drinking three cups of coffee daily may lower the risk of cardiovascular disease (CVD) mortality, Parkinson’s disease, type 2 diabetes, and various cancers in healthy individuals (11). However, the relationship between caffeine and bone density or osteoporosis remains inconclusive, with studies yielding conflicting results. The prevailing hypothesis suggests that caffeine competitively inhibits adenosine A2 receptors, thereby reducing bone formation and promoting bone resorption (12). Conversely, caffeine’s antagonism of A1 receptors may have the opposite effect, lowering osteoclast activity and indirectly enhancing bone formation (13). A systematic review by Wikoff et al. (14) concluded that adverse effects on bone health are only observed when daily caffeine intake exceeds 400 mg. Thus, further investigation into the mechanisms by which caffeine influences gut microbiota diversity and osteoporosis is crucial.

Aging is a multifactorial biological process closely linked to various chronic diseases and functional decline. It not only leads to reduced bone density and impaired physical function but also significantly impacts the diversity and functionality of the intestinal microbiota. Aging can be quantified using different biomarkers, such as the Klemera-Doubal Method (KDM), a biomarker-based approach for assessing physiological age; phenotypic age (PhenoAge), which reflects mortality risk; and the homeostasis disorder index (HD), which measures deviations from physiological balance (15). These indicators provide a comprehensive assessment of an individual’s aging from various perspectives.

Emerging evidence suggests that interventions targeting the brain-gut-bone axis may help reverse osteoporotic phenotypes. Yadav et al. reported that specific intestinal flora could prevent osteoporosis by enhancing bone synthesis through the Htr1b/PKA/CREB/cyclin signaling pathway, facilitated by reduced intestinal 5-HT synthesis (16). Additionally, a randomized controlled trial demonstrated that probiotics such as Bifidobacterium, Lactobacillus, Clostridium, Bacteroides, and Prevotella improved cortical thickness, trabecular volume, bone mineralization, and bone mineral density in the femur by modulating serum leptin levels (17). However, Arita et al. (18) noted that most current interventions focus on single bacterial strains, and the effects of different probiotic supplements are not yet robust enough for widespread application.

Recent research has also explored the relationship between “overall dietary indices” and osteoporosis. Common indices such as the Healthy Eating Index (HEI), Alternative HEI (aHEI), Mediterranean Diet Score (MDS), and Dietary Approaches to Stop Hypertension (DASH) have shown inconsistent associations with gut microbiota diversity and richness (19, 20). In response, Kase et al. (21) developed the Dietary Index for Gut Microbiota (DI-GM), a standardized tool for comprehensively evaluating diets that promote a healthy gut microbiota.

Currently, research on the DI-GM remains limited. In this study, we examined the relationship between the intestinal flora dietary index and osteoporosis using data from the NHANES 2007–2018. Additionally, we explored the roles of Phenotypic Age (PA), caffeine, and the KDM in mediating this relationship.

## Methods

### Data sources and study population

**Figure 1:**
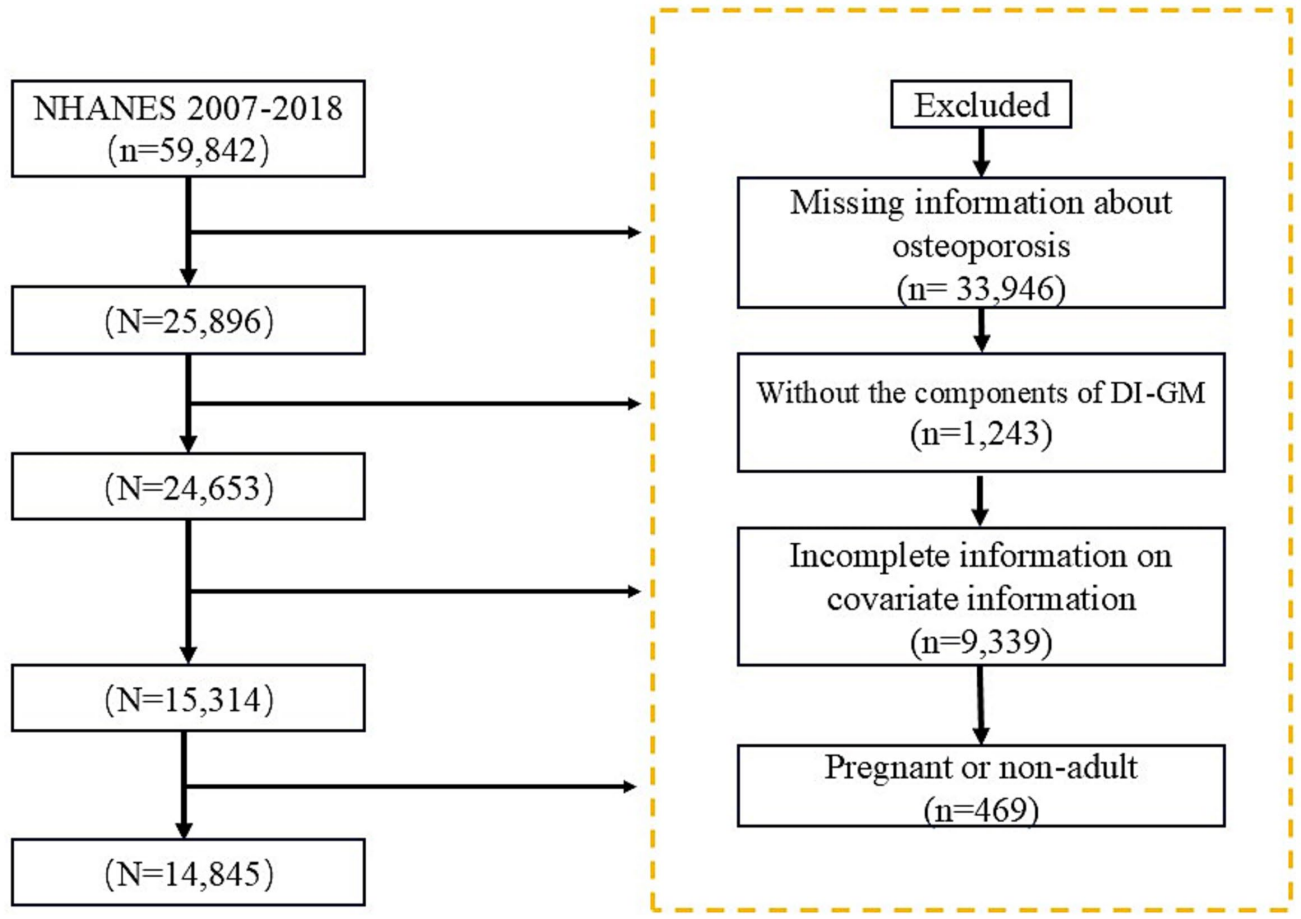
Flowchart of study population inclusion and exclusion criteria.

The National Health and Nutrition Examination Survey (NHANES) is a vital health and nutrition survey project conducted by the National Center for Health Statistics (NCHS) under the Centers for Disease Control and Prevention (CDC). Researchers can access the survey questionnaires, technical documentation, and analysis tools through the official CDC website: https://www.cdc.gov. For this study, we analyzed data from NHANES 2007–2018, ultimately including 14,845 participants who met the specified criteria, Figure 1.

### Exposure assessment

The DI-GM score incorporates 10 foods beneficial to gut health and 4 foods detrimental to it, providing a tool to evaluate the relationship between specific diets and gut microbiota (21, 22). Food and beverage data reported by participants during two 24-h dietary recall interviews were extracted using the U.S. Department of Agriculture (USDA) Food and Nutrient Database for Dietary Studies (FNDDS). To minimize potential bias from a single dietary record, the average of the two dietary recalls was used to calculate the DI-GM score. DI-GM employs sex-specific medians or fixed thresholds to score intake: 1 point is assigned for intakes of beneficial components above the median or unfavorable components below the median, resulting in a total score range of 0–14 points. Higher DI-GM scores indicate a dietary pattern with a more pronounced positive impact on gut microbiota. The specific calculation and scoring criteria are detailed in the study by Kase et al. (21).

### Mediating variables

In this study, the KDM and PA were employed to assess biological aging. Eleven key blood biochemical indices were measured using high-precision experimental techniques, such as enzyme kinetics and high-performance liquid chromatography (HPLC) (23–25). The relevant code for these analyses is available through the R package ‘BioAge’: https://github.com/dayoonkwon/BioAge. Daily caffeine intake was assessed using a 24-h dietary recall method, where participants reported all food and beverages consumed in the previous 24 h. To ensure comparability, all caffeine intakes were normalized for age.

### Outcome variable

Bone mineral density (BMD) of participants was measured in grams per square centimeter (g/cm^2^) using DXA with a QDR 4,500A fan-beam densitometer (Hologic Inc.) (26, 27). The diagnosis of osteopenia and osteoporosis followed the criteria established by the WHO. For this purpose, males and females aged 20 to 29 years were selected as the reference group. Participants were classified as having osteopenia if their BMD values were 1 to 2.5 standard deviations (SD) below the mean of the reference group, while osteoporosis was diagnosed when BMD values were more than 2.5 SD below the reference mean (28–30).

### Covariates

This study accounted for potential confounding variables that may influence osteoporosis (31). Demographic variables included age, gender, race, education level, and marital status. Socioeconomic variables were assessed using the poverty-to-income ratio (PIR). Additional covariates included hypertension, diabetes, cardiovascular disease (CVD), smoking status, drinking status, body mass index (BMI), and physical activity status. Smoking history was categorized into three groups based on past and current smoking behavior. Drinking status was classified into five categories according to drinking history and patterns. Furthermore, in addition to diabetes, prediabetic states such as impaired fasting glucose (IFG) and impaired glucose tolerance (IGT) were also included in the analysis.

### Statistical analysis

This study utilized the primary sampling unit (PSU) and stratification variables (Strata) to perform weighted analyses with multi-period weight adjustments (weights divided by 6). Continuous variables were presented as means and standard errors (SEs), and compared between groups using the Wilcoxon rank-sum test. Categorical variables were expressed as numbers (n) and percentages (%), and compared using chi-square tests.

In this study, the association between the DI-GM and osteoporosis was evaluated using multivariate logistic regression. The odds ratio (OR) was calculated to estimate the risk of osteoporosis. Model 1 included no covariate adjustments, model 2 adjusted for demographic variables (age, sex, race, education, and marital status), and model 3 accounted for age, sex, race, education, marital status, PIR, BMI, smoking, alcohol consumption, diabetes, hypertension, CVD, and physical activity status. The potential nonlinear association between DI-GM and osteoporosis was analyzed using RCS curves. Age-standardized incidence rates of osteoporosis and osteopenia were analyzed across different ethnic and age groups. Additionally, subgroup analyses were conducted based on variables such as gender, race, marital status, physical activity status, drinking habits, CVD, and hypertension to comprehensively explore epidemiological characteristics. Mediation analysis was performed using the “mediation” R package, and RCS curves were generated using the ggrcs package. All analyses were conducted using R version 4.2.3.

## Results

### Characteristics of participants

A total of 14,845 participants were included in the study and categorized based on their osteoporosis status, Table 1. The mean age of the participants was 41.03 years. Participants with osteoporosis had lower DI-GM scores, while those with osteopenia exhibited higher scores. Additionally, individuals with osteoporosis tended to have lower BMI, waist circumference, PIR, moderate physical activity rates, and probiotic and prebiotic intake. This group also had a higher proportion of females and non-Hispanic whites.

**Table 1:** Descriptive table of the study population by osteoporosis type.

Variable	Total	Normal	Osteopenia	Osteoporosis	*p*-value
Age	46.79 (0.27)	43.19 (0.28)	53.37 (0.39)	61.61 (0.84)	< 0.001
Sex (%)					< 0.001
Female	7,251 (49.57)	4,340 (44.51)	2,527 (59.01)	384 (68.50)	
Male	7,594 (50.43)	5,535 (55.49)	1,876 (40.99)	183 (31.50)	
Race/ethnicity (*n*, %)					< 0.001
Mexican American	2,245 (8.12)	1,535 (8.44)	640 (7.50)	70 (7.28)	
Non-Hispanic Black	2,854 (10.24)	2,320 (12.70)	490 (5.30)	44 (4.24)	
Non-Hispanic White	6,926 (69.40)	4,268 (66.98)	2,340 (74.32)	318 (75.03)	
Other Hispanic	1,401 (5.14)	941 (5.36)	410 (4.66)	50 (4.98)	
Other Race - Including Multi-Racial	1,419 (7.09)	811 (6.53)	523 (8.23)	85 (8.47)	
Moderate activity					0.002
No	8,682 (52.78)	5,661 (51.83)	2,635 (53.89)	386 (62.08)	
Yes	6,163 (47.22)	4,214 (48.17)	1,768 (46.11)	181 (37.92)	
Education level (*n*, %)					< 0.001
9-11th grade (Includes 12th grade with no diploma)	2,110 (10.54)	1,418 (10.40)	624 (10.99)	68 (9.34)	
College graduate or above	3,529 (30.36)	2,353 (30.50)	1,057 (30.60)	129 (25.22)	
High school graduate/GED or equivalent	3,458 (23.58)	2,295 (23.39)	1,013 (23.82)	150 (25.33)	
Less than 9th Grade	1,284 (4.21)	783 (3.89)	413 (4.23)	88 (10.56)	
Some college or AA degree	4,464 (31.31)	3,026 (31.82)	1,296 (30.36)	142 (29.55)	
Marital status (*n*, %)					< 0.001
Divorced	1,716 (10.70)	1,029 (9.21)	610 (13.55)	77 (15.37)	
Living with partner	1,185 (7.71)	918 (8.92)	248 (5.47)	19 (3.16)	
Married	7,811 (56.20)	5,150 (56.17)	2,381 (56.95)	280 (50.21)	
Never married	2,628 (18.25)	2,062 (21.00)	519 (13.06)	47 (8.68)	
Separated	476 (2.23)	327 (2.25)	136 (2.29)	13 (1.37)	
Widowed	1,029 (4.90)	389 (2.45)	509 (8.67)	131 (21.21)	
Drinking status (*n*, %)					< 0.001
Former	2,186 (11.64)	1,325 (10.56)	752 (13.53)	109 (16.74)	
Heavy	3,162 (22.19)	2,437 (25.11)	670 (16.91)	55 (9.98)	
Mild	5,219 (37.98)	3,413 (37.23)	1,614 (39.81)	192 (37.02)	
Moderate	2,403 (18.27)	1,672 (18.74)	669 (17.42)	62 (16.25)	
Never	1,875 (9.92)	1,028 (8.36)	698 (12.33)	149 (20.00)	
Smoking status (*n*, %)					0.050
Never	3,620 (24.42)	2,282 (23.38)	1,208 (26.72)	130 (25.28)	
Former	8,063 (55.63)	5,401 (56.11)	2,329 (54.59)	333 (55.13)	
Now	3,162 (19.94)	2,192 (20.51)	866 (18.69)	104 (19.59)	
Diabetes (*n*, %)					0.020
DM	2,527 (12.73)	1,591 (12.36)	819 (13.08)	117 (17.11)	
IFG	674 (4.49)	442 (4.45)	206 (4.48)	26 (5.34)	
IGT	559 (3.28)	337 (3.02)	187 (3.58)	35 (6.04)	
No	11,085 (79.50)	7,505 (80.17)	3,191 (78.86)	389 (71.51)	
CVD (*n*, %)					< 0.001
No	13,368 (92.30)	9,109 (93.97)	3,789 (89.34)	470 (84.72)	
Yes	1,477 (7.70)	766 (6.03)	614 (10.66)	97 (15.28)	
Hyperlipidemia (*n*, %)					< 0.001
No	4,412 (31.16)	3,158 (33.44)	1,127 (26.70)	127 (24.59)	
Yes	10,433 (68.84)	6,717 (66.56)	3,276 (73.30)	440 (75.41)	
BMI (kg/m^2^)	28.51 (0.09)	29.11 (0.10)	27.39 (0.12)	26.20 (0.34)	< 0.001
Waist (cm)	98.10 (0.23)	99.15 (0.28)	96.16 (0.36)	94.25 (0.89)	< 0.001
PIR	3.10 (0.04)	3.13 (0.04)	3.08 (0.06)	2.71 (0.11)	< 0.001
DI-GM	4.73 (0.02)	4.67 (0.03)	4.87 (0.04)	4.78 (0.08)	< 0.001

DI-GM, dietary index for gut microbiota. PIR, poverty income ratio. BMI, body mass index. CVD, cardiovascular disease. KDM, Klemera–Doubal Method. PA, phenotypic age. Continuous variables were expressed as weighted means and standard errors, while categorical variables were expressed as weighted percentages. For continuous variables, the p-value was based on the analysis of variance (ANOVA), and for categorical variables, the p-value was based on the chi-square test. A higher DI-GM score indicates a healthier gut microbiota.

### Association between DI-GM and osteoporosis

Weighted logistic regression analysis revealed that in Model 1, higher DI-GM scores were negatively associated with the risk of osteoporosis, Table 2. Compared to the Q1 group, the Q4 group had a significantly reduced risk of osteoporosis, with an OR of 0.768 (95% CI: 0.676–0.860). After adjusting for demographic variables in Model 2, the negative association between DI-GM and osteoporosis was attenuated in the Q2–Q4 groups. However, after adjusting for all variables in Model 3, the negative correlations among the four groups persisted. As the DI-GM score increased, the risk of osteoporosis decreased significantly, with ORs of 0.937 (95% CI: 0.883–0.995) for Q1 and 0.781 (95% CI: 0.693–0.869) for Q4.

**Table 2:** Multivariable logistic regression analyses for osteoporosis.

DI_GM	Model 1	*p*-value	Model 2	*p*-value	Model 3	*p*-value
Continuous	0.920 (0.880, 0.960)	<0.001	0.890 (0.850, 0.930)	<0.001	0.937 (0.883, 0.995)	0.0348
Q1(< 3)	Reference		Reference		Reference	
Q2(3,5)	0.853 (0.761, 0.945)	0.004	0.887 (0.801, 0.973)	0.016	0.879 (0.802, 0.956)	0.004
Q395,8)	0.812 (0.724, 0.900)	<0.001	0.845 (0.759, 0.931)	<0.001	0.829 (0.751, 0.907)	<0.001
Q4(≥8)	0.768 (0.676, 0.860)	<0.001	0.794 (0.703, 0.885)	<0.001	0.781 (0.693, 0.869)	<0.001

### Nonlinear association between DI-GM and osteoporosis

**Figure 2:**
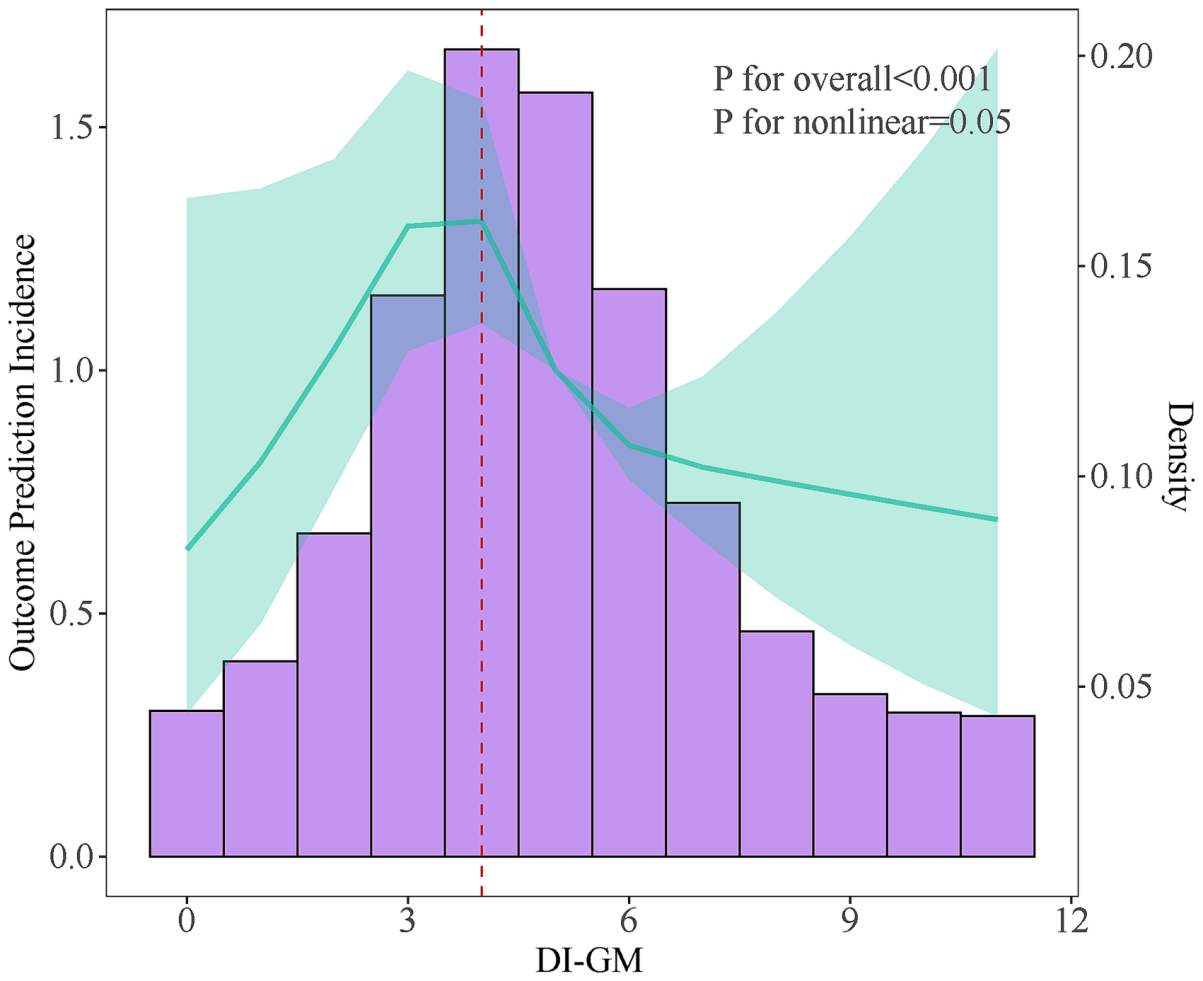
RCS curve depicting the nonlinear association between DI-GM and Osteoporosis Risk.

RCS curve and threshold effect analysis were employed to examine the nonlinear relationship between DI-GM and osteoporosis, Figure 2. After adjusting for all variables, the results indicated that as the DI-GM score increased, the overall risk of osteoporosis decreased. A nonlinear association between DI-GM and the risk of osteoporosis was identified, with a critical inflection point at 3.9, Table 3. Below this threshold, no statistically significant association was observed between DI-GM and osteoporosis. However, when the DI-GM score exceeded 3.9, a significant negative correlation emerged, with an OR of 0.875 (95% CI: 0.803–0.953).

**Table 3:** Dose–response relationship between DI-GM and osteoporosis.

Outcome	95%CI, *p*-value
Model 1 Fitting model by standard linear regression	0.93(0.877–0.986)0.015
Model 2 Fitting model by two-piecewise linear regression	
Inflection point	3.9
<3.9	1.084(0.92–1.288)0.344
>3.9	0.875(0.803–0.953)0.002
P for likelihood ratio test	0.05

DIGM and the changing trend of the incidence of osteoporosis and osteopenia.

**Figure 3:**
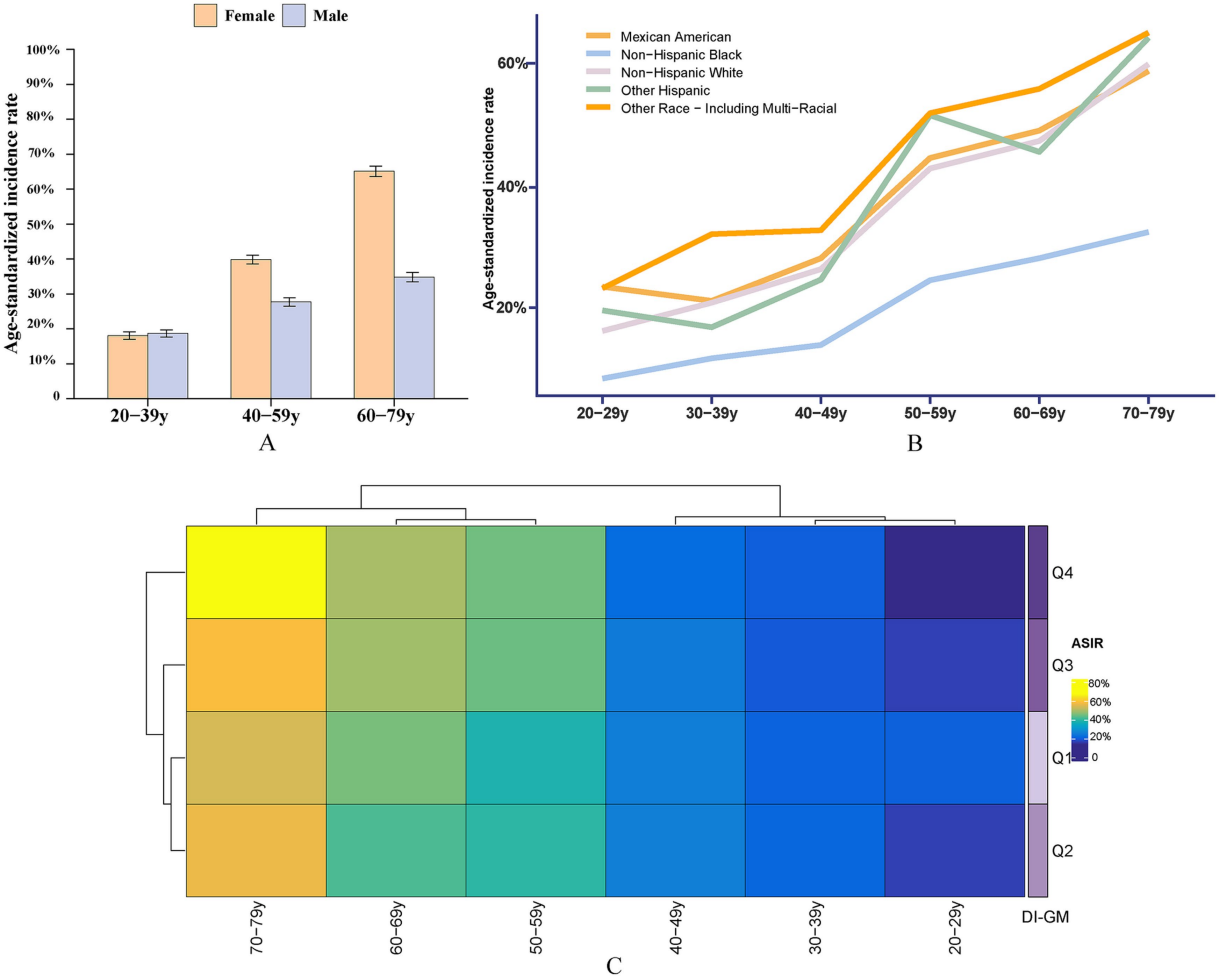
ASIR of osteoporosis and osteopenia across different demographic subgroups. **(A)** ASIR by sex across different age groups, highlighting the variation in osteoporosis prevalence between males and females. **(B)** ASIR by race/ethnicity, illustrating differences in osteoporosis incidence among Non-Hispanic White, Non-Hispanic Black, Hispanic, and other racial/ethnic groups. **(C)** ASIR by DI-GM quartiles: Q1 (<3), Q2 (3, 5), Q3 (5, 8), and Q4 (≥8), showing the relationship between DI-GM scores and osteoporosis incidence.

This study evaluated the trends in weighted Age-standardized incidence rate (ASIR) of osteoporosis and osteopenia across different DI-GM groups, ethnic groups, and age groups, Figure 3. The results revealed that the ASIR of osteoporosis and osteopenia was as high as 65.09% in women aged 60–79, while the ASIR in men aged 20–39 was 0.61% higher than in women, Tables 4, 5. Stratified analysis showed that the ASIR of osteoporosis and osteopenia among Non-Hispanic Blacks in all age groups was significantly lower than in other racial groups. Further analysis by dividing DI-GM into four groups indicated that before the age of 50, higher DI-GM scores were associated with lower ASIR of osteoporosis and osteopenia. However, after the age of 50, higher DI-GM scores were linked to a gradual increase in ASIR of osteoporosis and osteopenia.

**Table 4:** Age-standardized incidence rates across 10-year age groups.

Race	Age group	Age-standardized incidence rate	SE
Mexican American	20-29y	23.47662239	3.463389238
Mexican American	30-39y	21.12501901	2.146693714
Mexican American	40-49y	28.21211353	2.496165139
Mexican American	50-59y	44.88235911	2.819362544
Mexican American	60-69y	49.42958194	2.593433527
Mexican American	70-79y	59.32724413	3.335375305
Non-Hispanic Black	20-29y	8.20882314	1.306933431
Non-Hispanic Black	30-39y	11.57336144	1.275304338
Non-Hispanic Black	40-49y	13.78943569	1.347770858
Non-Hispanic Black	50-59y	24.55470035	1.774466036
Non-Hispanic Black	60-69y	28.22081555	2.220113311
Non-Hispanic Black	70-79y	32.56965949	2.156513538
Non-Hispanic White	20-29y	16.12369086	1.497537197
Non-Hispanic White	30-39y	20.82181077	1.408874631
Non-Hispanic White	40-49y	26.39394588	1.57059995
Non-Hispanic White	50-59y	43.13555837	1.60898121
Non-Hispanic White	60-69y	47.70184192	1.795950553
Non-Hispanic White	70-79y	60.46813857	1.674073941
Other Hispanic	20-29y	19.53986705	2.646220162
Other Hispanic	30-39y	16.74411209	1.895694393
Other Hispanic	40-49y	24.65845603	2.490348525
Other Hispanic	50-59y	51.98316607	3.179208468
Other Hispanic	60-69y	45.88888959	2.999884614
Other Hispanic	70-79y	64.86013992	5.04567774
Other Race - Including Multi-Racial	20-29y	23.21087709	2.705641977
Other Race - Including Multi-Racial	30-39y	32.22115545	2.954354772
Other Race - Including Multi-Racial	40-49y	32.86206198	3.008053921
Other Race - Including Multi-Racial	50-59y	52.33409425	4.990714038
Other Race - Including Multi-Racial	60-69y	56.3751331	5.087547756
Other Race - Including Multi-Racial	70-79y	65.71406129	5.080437348

**Table 5:** Age-standardized incidence rates by sex across 20-year age groups.

Sex	Age group	Age-standardized incidence rate	SE
Female	20-39y	18.0730274	1.075996139
Female	40-59y	39.81426722	1.259804113
Female	60-79y	65.0982165	1.499862485
Male	20-39y	18.68800692	1.00773047
Male	40-59y	27.71739778	1.188391685
Male	60-79y	34.80629448	1.328305912

### Subgroup analyses

**Figure 4:**
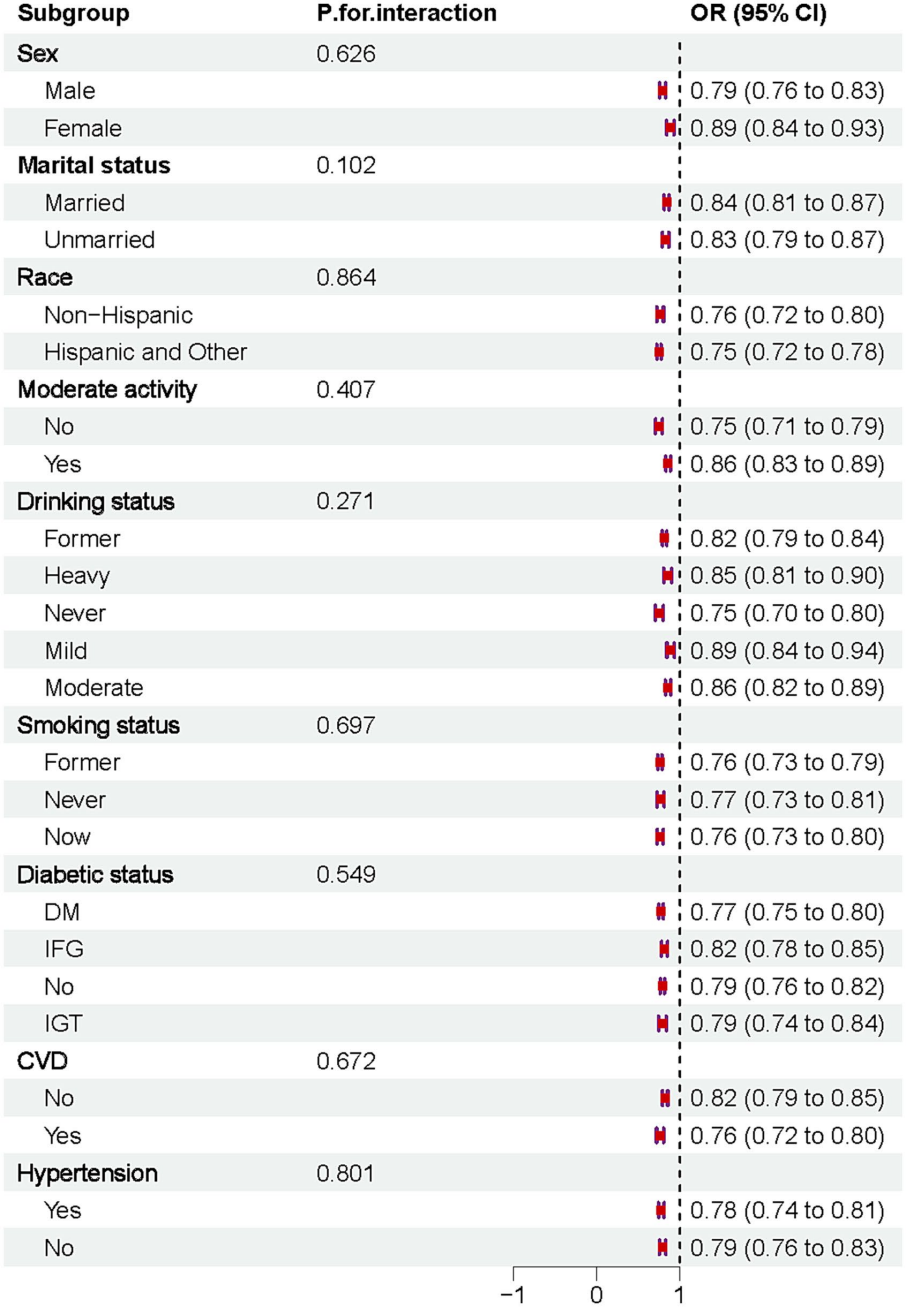
Forest plot of subgroup analyses for the association between DI-GM and osteoporosis risk.

The results indicated that DI-GM was negatively associated with osteoporosis across all subgroups. No significant interactions were observed after stratifying by sex, marital status, race, moderate physical activity, drinking habits, smoking status, diabetes, CVD, or hypertension, Figure 4.

### Mediation analysis

**Figure 5:**
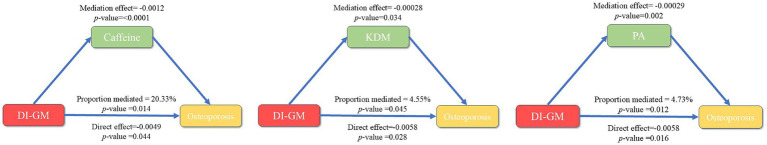
Mediation analysis of the association between DI-GM and osteoporosis: roles of caffeine, phenotypic age, and KDM.

This study employed mediation analysis to investigate the potential mediating roles of two biological aging indicators—PA and the KDM—as well as caffeine in the association between DI-GM and osteoporosis, Figure 5. The results revealed that the mediating effects of PA and KDM on the DI-GM-osteoporosis relationship were −0.00029 (*p* = 0.002) and −0.00028 (*p* = 0.034), respectively, with mediation percentages of 4.73 and 4.55%, Table 6. Caffeine demonstrated a stronger mediating effect on aging indicators, with a mediation percentage of 20.33% (*p* = 0.014).

**Table 6:** Results of mediation analysis: caffeine, phenotypic age, and KDM.

Caffeine	Estimate	95% CI lower	95% CI upper	*p*-value
Total effect	−0.006193	−0.011098	−0.001351	0.014
Mediation effect (average)	−0.001259	−0.00229	−0.000438	<0.0001
Direct effect (average)	−0.004933	−0.00988	0.000022	0.044
Proportion mediated (average)	0.203335	0.06198	0.872223	0.014
Phenotypic age				
Total effect	−0.006159	−0.011011	−0.001817	0.01
Mediation effect (average)	−0.000291	−0.000561	−0.00007	0.002
Direct effect (average)	−0.005867	−0.010808	−0.00154	0.016
Proportion mediated (average)	0.047328	0.00847	0.174895	0.012
KDM				
Total effect	−0.006162	−0.010995	−0.000907	0.02
Mediation effect (average)	−0.00028	−0.000581	−0.000024	0.034
Direct effect (average)	−0.005881	−0.010686	−0.000637	0.028
Proportion mediated (average)	0.04552	−0.002542	0.194533	0.045

## Discussion

This study identified a negative association between the DI-GM and osteoporosis. Since a higher DI-GM score reflects a diet that promotes greater intestinal microbiota diversity, maintaining microbiota diversity appears to have a protective effect in reducing the risk of osteoporosis. RCS analysis revealed a nonlinear association, with a critical turning point at 3.9. Mediation analysis further demonstrated that biological age and caffeine intake played significant mediating roles in the relationship between DI-GM and osteoporosis. Stratified analyses and interaction tests confirmed the robustness and stability of these findings.

With the aging global population, the number of individuals affected by osteoporosis in the European Union and the United States has surpassed 37.5 million, imposing a substantial burden on society and families (32). Although medications such as bisphosphonates, teriparatide, and denosumab can effectively treat osteoporosis, early diagnosis and prevention remain significant challenges in clinical practice (33). In recent years, the relationship between intestinal flora and osteoporosis has garnered significant attention from scholars. Research has shown that certain functional foods, such as astragalus polysaccharides, can mitigate refractory osteoporosis by reducing osteocalcin and TNF-*α* levels, likely through modulation of five key bacterial species (*uncultured_bacterium_f_Ruminococcaceae*, *Alloprevotella*, *Ruminococcaceae_UCG-014*, *Blautia*, and *Lactobacillus*) (34). Kenichi et al. demonstrated that dietary fructo-oligosaccharides and glucomannan reduce bone resorption by alleviating systemic inflammation (35). Similarly, Zhang et al. found that folic acid supplementation from B vitamins promotes the expression of LCA and TGR5, thereby preventing bone loss associated with high body fat (36). Diet is one of the simplest, most cost-effective, and traditional methods for regulating intestinal flora composition and function, improving intestinal barrier integrity and immune system health in a short period (37). Collectively, these studies indicate that improving dietary structure or supplementing specific nutrients can prevent osteoporosis and osteopenia by influencing bone metabolism through multiple pathways. This aligns with our view that dietary modifications favoring diverse gut microbiota could positively impact osteoporosis outcomes.

Bezawit et al. (21) and his team developed a novel dietary index, DI-GM, to reflect changes in intestinal microbiota diversity, short-chain fatty acid (SCFA) production levels, and specific bacterial counts. However, the relationship between DI-GM and osteoporosis remains unclear. Our study demonstrated that an increase in DI-GM score was significantly associated with a reduced risk of developing osteoporosis. The beneficial gut health indicators included in DI-GM are improvements in *α*-diversity and *β*-diversity, balance in the Firmicutes/Bacteroidetes ratio, and elevated levels of total SCFAs, including butyrate, acetate, propionate, and isobutyrate (21, 38). The diverse gut microbiota encompassed by DI-GM are widely recognized for their important influence on bone health.

Preclinical studies have shown that supplementation with lactic acid bacteria significantly increases trabecular bone volume fraction in mice, although it does not significantly affect trabecular bone number or thickness (39). In clinical studies, supplementation with Faecalibacterium and Roseburia inhibits bone resorption and promotes bone density by activating the GPR43 receptor through SCFA production, particularly butyrate (40). Additionally, supplementation with Bifidobacterium and Lactobacillus has been shown to reduce osteopenia caused by bone resorption by suppressing immune responses (41). *Akkermansia muciniphila* supplementation positively influences bone density by regulating fat metabolism and reducing systemic inflammation (42). Notably, Prevotella species can alleviate bone inflammation by expressing Foxp3 in Treg cells, while products of Anaerostipes hadrus may affect bone formation by modulating the interaction between gut microbiota and the immune system (43). These findings, along with the results of this study, suggest that a diverse gut microbiota helps maintain intestinal homeostasis and supports bone health through multiple metabolic pathways.

This study identified a nonlinear association between the DI-GM score and osteoporosis using RCS curve and threshold effect analysis. A significant negative correlation between DI-GM and osteoporosis was observed only when the DI-GM score exceeded 3.9 (OR: 0.875, 95% CI: 0.803–0.953). An analysis of osteoporosis and osteopenia incidence trends revealed heterogeneity in ASIRs across different age groups. This highlights the complex interactions between age, race, and DI-GM scores, suggesting that universal dietary recommendations are not feasible. Instead, personalized dietary strategies should account for age and racial differences. Before the age of 50, a dietary pattern with a higher DI-GM score is recommended to promote intestinal microbiota diversity and support bone health. After the age of 50, however, the DI-GM score may need to be moderately reduced to avoid potential adverse effects.

The mechanism by which DI-GM affects osteoporosis requires further investigation. Numerous studies have explored the roles of dietary patterns, gut microbiota composition, mitochondrial dysfunction, and oxidative stress in bone metabolism (44, 45). One key finding is that biological age and caffeine mediate the association between DI-GM and osteoporosis. Evidence suggests that biological aging not only reduces the diversity of intestinal flora but also compromises intestinal barrier function, allowing bacterial toxins such as lipopolysaccharide (LPS) to leak into the bloodstream, thereby inducing “inflammatory aging (46, 47).” Excessive caffeine intake is widely recognized as an independent risk factor for osteoporosis, affecting bone metabolism through various mechanisms (48). Berman et al. reported that caffeine-induced oxidative stress damages osteoblasts and the bone matrix while stimulating osteoclast activity, accelerating bone breakdown (13). Additionally, caffeine has been linked to increased calcium loss. Ohta et al. (11) found that individuals consuming more than 400 mg of caffeine daily exhibited significantly lower bone density and markedly increased urinary calcium excretion.

Interestingly, gut microbiota play a critical role in caffeine metabolism. The intestinal flora can regulate caffeine levels in the body by modulating its absorption and excretion (49). Gu et al. (50) demonstrated that alterations in the Firmicutes-to-Bacteroidetes ratio influence the intestinal absorption of caffeine, thereby affecting its concentration in the bloodstream. An intervention study found that Bifidobacterium and Lactobacillus reduced caffeine absorption efficiency and its accumulation in the body by altering intestinal pH and digestive enzyme activity, mitigating its negative impact on bone metabolism (51). Furthermore, an imbalanced gut microbiota can alter the activity of liver metabolic enzymes, such as CYP1A2, slowing caffeine metabolism and leading to its accumulation, thereby increasing osteoporosis risk (52). Thus, adopting a diet that promotes gut health may help slow aging, reduce caffeine absorption, and ultimately lower the risk of osteoporosis.

### Advantages and limitations

This study has several limitations. First, food intake data were collected through 24-h recall interviews or telephone interviews, which are subject to reporting errors. Second, respondents may have been influenced by social desirability bias, leading to an underestimation of the intake of unhealthy foods, such as high-sugar and high-fat items. Finally, as a cross-sectional study, causality cannot be established, meaning the observed associations may be confounded by unmeasured factors such as lifestyle, genetic predisposition, or psychological status.

Nevertheless, the DI-GM used in this study is a novel indicator developed from intervention research. Unlike a single biomarker (e.g., *β*-glucuronidase activity or SCFA levels), DI-GM integrates 14 foods and nutrients that are closely associated with intestinal health, providing a more comprehensive measure of the overall dietary impact on gut microecology. Future longitudinal studies and intervention trials are necessary to confirm the causal relationship and further elucidate the potential mechanisms through which DI-GM reduces the risk of osteoporosis.

## Conclusion

This study revealed, for the first time, a significant negative association between the DI-GM score and osteoporosis risk. Mediation analysis indicated that biological age and caffeine intake play important roles in this relationship. Age-standardized incidence rate analysis showed that osteoporosis ASIR was highest among women aged 60–79 years (65.09%) and significantly lower in the Non-Hispanic Black group compared to other racial groups. Collectively, these findings suggest that adopting a dietary pattern promoting intestinal microbiota diversity may help reduce osteoporosis risk. Personalized dietary strategies should be tailored for individuals based on race and age group to maximize their effectiveness.

## Data Availability

The original contributions presented in the study are included in the article/supplementary material, further inquiries can be directed to the corresponding authors.
